# A QUALITATIVE STUDY OF ADULT PROTECTIVE SERVICES PRACTITIONERS RESPONDING TO CASES OF ELDER ABUSE AND SELF-NEGLECT

**DOI:** 10.1093/geroni/igae098.2918

**Published:** 2024-12-31

**Authors:** Andie MacNeil, Erin Salvo, David Burnes

**Affiliations:** University of Toronto, Toronto, Ontario, Canada; Public Consulting Group, Portland, Maine, United States; University of Toronto, Toronto, Ontario, Canada

## Abstract

Adult Protective Services (APS) practitioners play a critical role in supporting older adults experiencing elder abuse and self-neglect (EASN), however, very little research has examined their experiences, from their perspectives. The purpose of this study was to examine the experiences of APS practitioners responding to allegations of EASN. Using a descriptive phenomenological qualitative methodology, semi-structured interviews were conducted with APS practitioners (n = 14) from the state of Maine. To enhance trustworthiness, two independent assessors analyzed transcript data to code transcripts into themes. Two domains, each with various subthemes, were identified: (1) rewarding elements of role and (2) challenging aspects of role. The findings of this study emphasize how APS practitioners are motivated by their capacity to help elicit positive change in the lives of their clients and support the well-being of older adults experiencing EASN. However, APS practitioners must navigate numerous challenges and barriers in their role, including time constraints, high and complex caseloads, limited resources, and broader misconceptions on APS. These findings highlight the importance of addressing these stressors to support the well-being of APS practitioners, which, in turn, can help support the vulnerable older adults they serve.

